# Good Financial Grant Practice: A Tool for Developing and Demonstrating Institutional Financial and Grant Management Capacity in Global Health

**DOI:** 10.1093/cid/ciab768

**Published:** 2021-11-25

**Authors:** Harry J Harste, Genevieve Kiff, Iruka N Okeke, Akindele O Adebiyi, K L Ravikumar, Geetha Nagaraj, Jolaade J Ajiboye, Erik C D Osma Castro, Elmer Herrera, David M Aanensen, NIHR Global Health Research Unit on Genomic Surveillance of Antimicrobial Resistance, Khalil Abudahab, Monica Abrudan, Silvia Argimón, Mihir Kekre, Dawn Muddyman, Ben Taylor, Anthony Underwood, Nicole Wheeler, David Sophia, Pilar Donado-Godoy, Johan Fabian Bernal, Alejandra Arevalo, Maria Fernanda Valencia, Varun Shamanna, Vandana Govindan, Akshata Prabhu, D Sravani, M R Shincy, Steffimole Rose, K N Ravishankar, Anderson O Oaikhena, Ayorinde O Afolayan, Erkison Ewomazino Odih, Celia Carlos, Marietta L Lagrada, Polle Krystle V Macaranas, Agnettah M Olorosa, June M Gayeta

**Affiliations:** 1 Oxford Big Data Institute, University of Oxford, Oxford, United Kingdom; Wellcome Genome Campus, Hinxton, United Kingdom; 2 Oxford University Clinical Research Unit, Hanoi, Vietnam; African Academy of Sciences, Nairobi, Kenya; 3 Department of Pharmaceutical Microbiology, University of Ibadan, Ibadan, Nigeria; 4 College Research and Innovation Management, College of Medicine, University of Ibadan, Nigeria; 5 Central Research Laboratory, Kempegowda Institute of Medical Sciences, Bengaluru, India; 7 AGROSAVIA (Corporación Colombiana de Investigación Agropecuaria), Bogotá, Colombia; 8 Antimicrobial Resistance Surveillance Reference Laboratory, Research Institute of Tropical Medicine, Manila, Philippines

**Keywords:** GFGP, Good Financial Grant Practice, research funding, grant management, governance

## Abstract

The administration and governance of grant funding across global health organizations presents enormous challenges. Meeting these challenges is crucial to ensuring that funds are used in the most effective way to improve health outcomes, in line with the United Nations’ Sustainable Development Goal 3, “Ensure healthy lives and promote well-being for all at all ages.” The Good Financial Grant Practice (GFGP) Standard (ARS 1651) is the world’s first and, currently, only international standard for the financial governance and management of grant funding. Through consensus building and global harmonization between both low- and middle-income and high-income country players, the GFGP Standard has achieved a leveling impact: GFGP applies equally to, and can be implemented by, all types of organization, regardless of location, size, or whether they predominantly give or receive funding.

GFGP can be used as a tool for addressing some of the challenges of the current funding model. Here, we describe our experiences and lessons learned from implementing GFGP across 4 diverse research institutions in India, Nigeria, Colombia, and the Philippines as part of our National Institute for Health Research Global Health Research Unit on Genomic Surveillance of Antimicrobial Resistance.

Unprecedented funds are being pledged and used to support public health research and services globally. In 2004, global health funding was around $14 billion, and this increased rapidly, largely due to initiatives such as the Bill & Melinda Gates Foundation and the US government’s AIDS Initiative [1, 2]. In 2015, development assistance for health (DAH) funding alone accounted for around $19 billion. Particular importance was placed on the 31 low-income countries where external sources of funding accounted for 33% of total health spending on average in 2015, with this proportion increasing over time in absolute terms [3].

The administration and governance of funds across a wide geographical range and number of global health organizations involve significant challenges. Meeting these challenges is crucial to ensuring funds are used most effectively to meet their intended aim of improving health outcomes, in line with the United Nations’ Sustainable Development Goal (SDG) 3, “Ensure healthy lives and promote well-being for all at all ages” [4].

Developing institutional capacity to receive and administer funding at grantee organizations is a key component in ensuring that institutional structures are effective at managing large scientific grants to achieve intended research aims [5]. Similarly, the role of effective governance on health outcomes is crucial. Research has shown that poor governance and corruption contribute more to antibiotic resistance rates than antibiotic usage volumes, demonstrating the importance of governance in supporting effective health outcomes [6].

Here, we describe the work undertaken as part of the National Institute for Health Research (NIHR) Global Health Research Unit (GHRU) on Genomic Surveillance of Antimicrobial Resistance in implementing the Good Financial Grant Practice (GFGP) Standard as a method of addressing some of the challenges of the funding model.

## CHALLENGES AND DEVELOPMENTS IN THE FUNDING MODEL

### Inadequate Institutional Capacity Support

Despite the unprecedented flow of global health funding into low- and middle-income countries (LMICs), there are often significant gaps in physical infrastructure, human capital, governance, and management structures (including procurement systems) in LMIC research institutes [5, 7–10]. These gaps include a lack of institutional financial and grant management capacity, creating major challenges around the administration of grant funding.

A lack of funding to support institutional capacity can create a vicious cycle, one in which LMIC organizations are starved of support for indirect costs (also referred to as “overheads,” ie, expenses that are not directly attributable to a specific project or grant, such as utilities and administrative staff costs) [5, 11]. Continual, inadequate indirect cost support from grantors means grantees may increasingly struggle to function effectively, but they are often reluctant to ask for more support in case the request is detrimental to future funding applications and collaborations. This can create a perpetuating cycle of indirect cost underfunding [11–13]. Irrespective of how well a particular project is designed and implemented, if the organizational capacity to effectively manage its financial resources is not in place, there is likely to be a significant impact on the delivery and sustainability of the project [14].

However, adequately funding indirect costs alone is not always enough to develop institutional financial and grant management capacity. Capacity building support, for example, by establishing and training a successful grants administration office, is sometimes also required to enable institutions to effectively use indirect cost funding, with increasing awareness that this support is a requisite component for managing grant funding [15]. For example, in 2018, the NIHR announced supplementary financial assurance funding to their Global Health Research awards for specific financial capacity-building activities with LMIC partners [16]. The NIHR has developed this approach further by allowing these costs to be included within direct program costs for their awards commencing in 2021 [17].

### Ineffective Governance

Poor governance and corruption are fundamental challenges for the administration of grant funding, and they represent a significant barrier to universal health coverage by preventing funding from reaching intended recipients and thereby directly impacting research projects and SDG 3. Estimating the scale of this problem is challenging, but it constitutes a significant issue in global health and beyond [18, 19]. In addition, corruption has been shown to be a key socioeconomic factor in explaining antibiotic resistance rates [6].

Effective governance within research institutions is crucial to ensure effective decision making, at a programmatic level and more widely, and to improve both accountability and trust within the institution and across its personnel. The development of effective governance through the development of institutional capacity is also an important component in the fight against corruption [18].

### Ineffective and Inefficient Due Diligence

The current process for granting and receiving global health funding involves grantors, often high-income country (HIC)–based funders, asking multiple disparate due diligence questions of grantees (due diligence is an organizational review that is completed before entering into an agreement with that organization). There is often a lack of clarity and understanding of what is being asked for and what represents best practice, with due diligence requirements usually differing between funding agencies and institutions. There is also a power imbalance in this relationship, where grantors are requesting information before either programmatic activity begins or funds are transferred, putting onerous requirements on grantees in terms of staff time and cost of compliance. For example, in 2018 and 2019, a Nigerian university had more than 20 due diligence requests from 16 global funders, including UNICEF, the African Academy of Sciences, the International Development Research Centre, UK NIHR, and UK Research and Innovation Global Challenges Research Fund.

There is a clear need for the adoption of a standardized and more equitable approach to due diligence, through which LMICs and HICs contribute to the development of a due diligence framework that works for and is better understood by both grantors and grantees. This will improve the identification of key risks and reduce the duplication of effort, administrative burden, and costs for all players [20].

Various existing initiatives attempt to standardize an approach to due diligence. However, their scope is generally limited to specific sectors or countries, and they do not represent a globally harmonized approach [21].

### The GFGP Standard

The GFGP Standard (ARS 1651) is the world’s first and, currently, only public international quality standard for the financial governance and management of grant funding [22]. The GFGP Standard represents a paradigm shift in approach, using consensus building and global harmonization between LMIC and HIC players.

The standard was developed by the African Academy of Sciences in Nairobi, Kenya, in collaboration with global grantors and grantees from 22 countries across eastern, western, and southern Africa. This involved some of the world’s largest public and private sector funders, including those that provided funding, Wellcome, UK Research and Innovation, UK Department of Health and Social Care, the IKEA Foundation, the European and Developing Countries Clinical Trials Partnership, and other collaborators, the African Union; UK Foreign, Commonwealth, and Development Office (formerly DfID); the African Organisation for Standardisation; and the New Partnership for Africa’s Development and Coordinating Agency [22].

The initiative was developed through a series of interactive workshops, with active participation from LMIC grantees and global grantors. The GFGP Standard is uniquely designed when compared with other standards or due diligence tools. The engagement of grantees and grantors from a wide range of countries and risk profiles in the design and development of GFGP make it possible to apply the standard to effectively cover the key financial and grant management risks that affect organizations and directly impact programmatic delivery.

**Figure 1.**
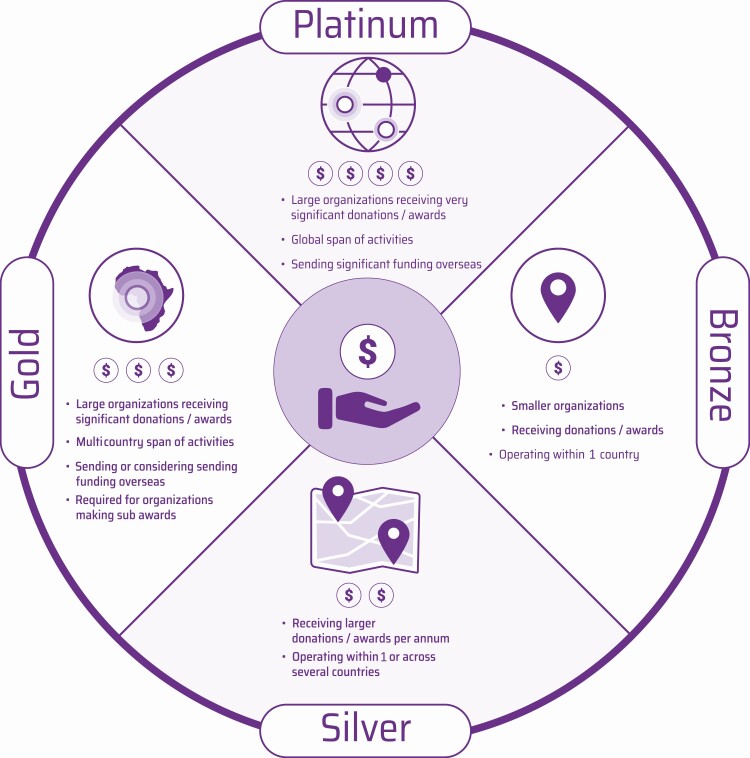
One standard accommodates all. The 4 levels of the GFGP Standard—platinum, gold, silver, and bronze—detailing the types of organization for which each level is intended. Adapted with permission from the original, by the Global Grant Community, African Academy of Sciences.

The GFGP Standard is made up of 4 practice areas: financial management, human resources, procurement, and governance. Adherence follows a series of steps. First, organizations self-assess against 1 of the scheme’s 4 tiers (bronze, silver, gold, and platinum); each tier is applicable to different types of organizations (Figure 1). The identification of an appropriate tier is a crucial step in the process, as the tiers do not represent an incremental continuum but are designed for organizations of differing sizes and complexities. Self-assessment involves completing a precertification assessment through an online portal, where between 70 (bronze) and 300 (platinum) clauses are completed and supporting procedural (bronze), process (silver), and policy (gold/platinum) documentation is uploaded. An organization can self-assess against GFGP at an institutional or departmental level. This decision is based on how the organization administers its grant funding, either by central administration function or by self-contained departmental administration function.

Second, once organizations have self-assessed, they can request to be certified (audited) against the GFGP Standard by a licensed auditor (certifying body [CB]). CBs will review the adequacy of the procedures, processes, and policies in addressing key organizational risks and the practical implementation of the documents to address those risks (over at least the previous 3 months). If the CB assesses the organization as fully compliant, they will issue a certificate of compliance along with an accompanying audit report detailing findings. This process is supported by an online portal, where grantees can share their certificates and report findings with other grantors. The GFGP Standard, online portal, certification scheme, and CBs are collectively known as the Global Grant Community (GGC) [22].

### Developing Institutional Capacity

To avoid accreditation disadvantaging low-resource settings, requirements need to be transparent and ordered, making it possible for institutions that have gaps in capacity to develop a road map to meeting requirements [23]. The GFGP Standard provides a clear framework for improving institutional financial and grant management capacity.

When completing the GGC portal questionnaire, an organization can identify and track gaps in their current compliance. If this is the case, an implementation plan toward full GFGP compliance should be designed and undertaken. This is likely to include a series of recommendations around designing new documentation or refining existing documentation and then embedding this in organizational practice. Organizations may look to engage an external GFGP implementation consultant or employ a financial and grant management specialist to identify gaps and design effective implementation plans. However, this may be a challenge in resource-constrained settings, where funders are unable or unwilling to provide funding. As the number of GFGP-certified organizations increases, there appears to be scope for cross-institutional mentoring in the initiation phase of the implementation process.

Once an organization is confident that their documentation is adequate and has been practically implemented, a CB can be engaged to undertake the GFGP certification (audit). Certification gives the organization international recognition and gives funders and key stakeholders confidence in the effectiveness of institutional financial and grant management.

### Enhancing Institutional Governance

There is a specific focus in the GFGP Standard on improving governance and therefore trust and accountability within organizations. In the development of the GFGP Standard, the importance of effective governance was recognized in the creation of a separate section on governance (section 8) that contains 4 subsections (general governance, audit, grant compliance, and risk management).

For example, within the risk management subsection of the standard (section 8.4), there is a focus on the mechanisms that organizations put in place to enable individuals to confidentially report incidents of possible fraud, corruption, and bribery. The CB will check to see whether the procedural steps around disclosing fraud, bribery, and corruption have been followed and can be demonstrated. In this example, any significant issues identified by the CB around governance would need to be addressed by the organization before the CB issues a certificate of GFGP compliance.

### Effective and Efficient Due Diligence

Through consensus building of grantors and grantees from HICs and LMICs during the development process, the GFGP Standard has a leveling impact, which is significant in the due diligence process, when grantors request that grantees comply with certain requirements. This leveling impact is also created by a “one standard” approach, whereby GFGP applies equally to, and can be implemented by, all types of organization (Figure 1).

Organizations can use GFGP in both pre- and post-award due diligence by requesting that another organization complete a self-assessment questionnaire on the GGC online portal against 1 of the standard’s tiers. The requesting organization can then review the self-assessment responses on the GGC portal and follow up with any supplementary questions.

GFGP certification simplifies the due diligence process significantly. The GFGP certificate and accompanying report become evidence of compliance across the 4 practice areas, providing the assurance the grantor needs that the grantee organization is compliant with an international quality standard in grant management. The grantor may ask supplementary assurance questions. To support grantors in identifying and asking supplementary questions, the GGC has also designed a set of non-GFGP assurance questions around safeguarding, programmatic delivery, legal and compliance, and health and safety [22].

Although there may be a significant initial investment of time and resources to achieve full GFGP certification, the benefits of simplified due diligence are considerable, with less time and money spent on financial and grant compliance and more resources available to support programmatic delivery.

## IMPLEMENTING GFGP IN GLOBAL HEALTH ORGANIZATIONS

As part of the NIHR GHRU on Genomic Surveillance of Antimicrobial Resistance, we have implemented the GFGP Standard with our 4 partners: a private–public nonprofit research organization in Colombia (AGROSAVIA[Corporación Colombiana de Investigación Agropecuaria]), a medical college in India (Central Research Laboratory, Kempegowda Institute of Medical Sciences [CRL KIMS]), a large public university in Nigeria (University of Ibadan [UI]), and a public hospital in the Philippines (Research Institute for Tropical Medicine [RITM]).

GFGP was principally implemented as a method of building and demonstrating sustainable financial and grant management capacity to complement the scientific capacity that has been developed through whole-genome sequencing (WGS) implementation [24, 25]. WGS is becoming the method of choice for applications that include pathogen surveillance, biomarker discovery, and host response to disease, which have to be implemented in situ. WGS approaches provide the opportunity to flexibly add on new capacities as well as to leapfrog earlier roadblocks to science and health advancement in LMICs [26–29]. In contrast to many other global health endeavors that focus heavily on staff and patients, implementing WGS additionally entails large purchase contracts, agile procurement systems, and predominantly high-level staffing. For these reasons, it is especially vulnerable to gaps in GFGP compliance, particularly across human resources and procurement.

In 2019, we worked with the 4 institutes to complete the GFGP self-assessment questionnaire on the GGC portal. Diagnostic assessment visits were then performed at each partner site, with the Nigeria assessment visit performed in partnership with Humentum [30]. These visits focused on assessing current levels of GFGP compliance and providing organizational implementation plans to reach full GFGP compliance. The GFGP implementation lead worked remotely in collaboration with partner GFGP leads to implement the plans and reach full GFGP compliance. Each partner is currently at a different stage of implementation.

AGROSAVIA methodically implemented all the gold tier recommendations made following a diagnostic assessment visit in 2019 and has now successfully completed the certification process, becoming GFGP certified at gold tier on 7 September 2021. The 2019 assessment visit estimated AGROSAVIA’s overall compliance with the GFGP gold tier at around 80%. Although AGROSAVIA had advanced systems and practices in place across all GFGP practice areas, international grant funding was a relatively new funding stream for the organization. Consequently, the development and formalization of the international elements of the standard, such as procedures and policies on exchange rates, indirect costs, and recording time/effort on grants, were critical to enable AGROSAVIA to fully comply with the GFGP gold tier requirements.

RITM has established a GFGP committee but has yet to implement the bronze tier recommendations. This is largely due to the current challenges faced by RITM in being the principal coronavirus disease 2019 (COVID-19) testing site in the Philippines.

UI has also established a GFGP committee to address the bronze tier recommendations; however, this will require a transformational change within UI that may take a number of years to implement. Reaching GFGP certification is likely to be a challenging process for UI. However, given the significant number of funders this public university works with and the large number of principal investigators served by the grant management platforms, and therefore the volume of due diligence requests and funding administered, certification is likely to have significant organizational benefit to the university. We intend to continue to work in partnership beyond the life of this project to address these issues.

CRL KIMS is now fully GFGP-certified, as outlined in the following case study.

### Case Study: The World’s First GFGP Certification

CRL KIMS is a small research laboratory with a self-contained administration function. It has internationally certified (International Standards Organization [ISO] 1589:2012) research practices and facilities, but the level of grant management practices was not consistent. Prior to 2019, some financial and operational controls were in place, but these were not consistently applied or documented. A diagnostic assessment visit performed in 2019 identified overall compliance with the GFGP Standard at the bronze tier of around 65%.

**Figure 2.**
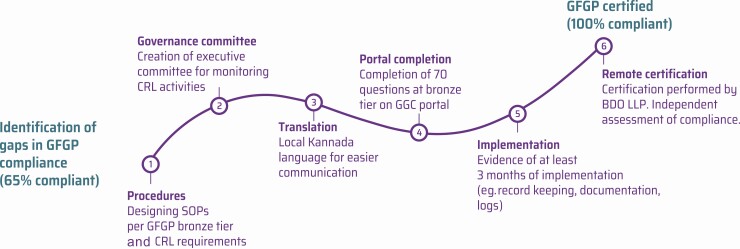
CRL KIMS’ journey to certification. The 6 steps taken by CRL KIMS in India, from identification of gaps in compliance to full GFGP certification. Abbreviations: BDO LLP; CRL KIMS, Central Research Laboratory, Kempegowda Institute of Medical Sciences; GFGP, Good Financial Grant Practice; GGC, Global Grant Community; SOP, standard operating procedure.

Six steps were taken in implementing GFGP at CRL KIMS, from the identification of gaps in GFGP compliance through to successful GFGP certification (Figure 2). As a partnership, we drafted a customized manual of standard operating procedures (SOPs), using a similar format to CRL KIMS’ laboratory (ISO) documentation. We created 21 individual SOPs, ensuring that the GFGP bronze tier requirements were met and that the procedures satisfied CRL KIMS’ organizational needs. A governance committee was also constituted to ensure that CRL KIMS had adequate structures for effective governance in line with the GFGP Standard.

Once these procedures were finalized, they were translated into the local language, Kannada, to ensure effective communication among all team members who had different levels of ability in the English language. The translation process was critical for ensuring that all members of the team were comfortable with the detailed procedures.

The project then entered the practical implementation phase, focused on embedding the procedures and demonstrating at least 3 months of implementation to the CB (BDO LLP) [31]. This work culminated in the production of a detailed electronic certification file for the CB for remote delivery (driven by COVID-19 restrictions).

CRL KIMS was GFGP certified on 15 June 2020, with 2 minor nonconformities (noncompliances) noted in the audit findings report.

Certification has brought multiple benefits to CRL KIMS by providing a best-practice structure to the administration function and developing staff knowledge and understanding of financial and grant management. Since certification, CRL KIMS’ organizational credibility has been enhanced, and this has facilitated and expedited the formalization of relationships with external partners and funders. For example, a multinational vaccine company and an Indian private diagnostics company have recently formalized partnerships with CRL KIMS. Obtaining GFGP certification played a major role in reducing the time taken to formalize these agreements and release funds, by 40%–50% (from between 10 to 12 weeks to around 6 weeks), when compared with similar agreements made prior to becoming GFGP certified.

**Figure 3.**
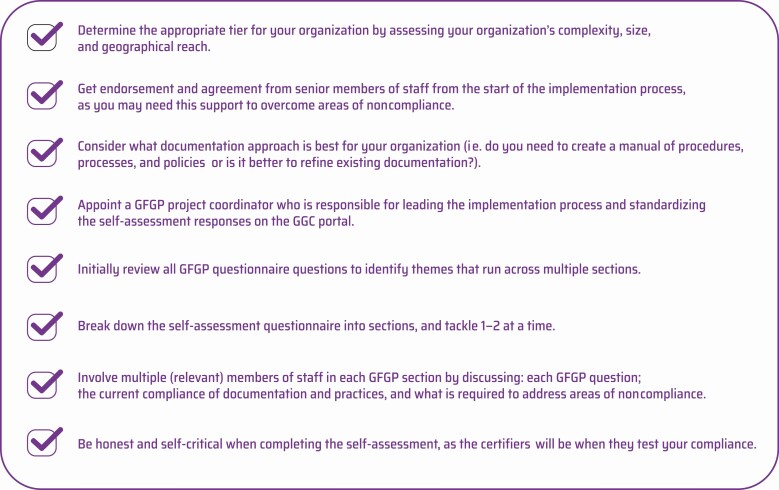
Practical tips for implementing GFGP. Abbreviations: GFGP, Good Financial Grant Practice; GGC, Global Grant Community.

There were a number of useful practical lessons learned from implementing GFGP at CRL KIMS and across our GHRU partners (Figure 3).

### Refinements to the GFGP Standard

While the benefits of implementing GFGP can be considerable for institutions, our practical implementation has highlighted some areas that require refinement and enhancement.

First, some of the GFGP questions appear repetitive. For example, there is some crossover in bronze (procedural) and silver (process) tier questions. This contributes to a doubling of the number of questions between bronze and silver, from around 70 to 140, but not a doubling of required effort to comply with the standard. Therefore, a review of bronze and silver requirements would provide greater distinction between the 2 tiers and remove any repetition. This would also ensure that the silver tier better represents more complex organizations, such as research organizations that manage large grant awards, while enabling smaller organizations, such as community-based organizations, to comply with a streamlined bronze tier.

Second, although compliance with the GFGP Standard will have an overall positive impact on programmatic delivery for institutions, such as by ensuring appropriate human resource practices to support high-level staffing requirements, there are some areas of the standard where amendments would enhance programmatic delivery. For example, while the procurement section of the standard requires detailed processes and policies to be implemented, the standard does not specifically refer to the need for timely procurement. Inclusion of a clause to this effect would strengthen the standard by addressing the operational risk of sluggish implementation of the procurement practices and the subsequent potential impact on programmatic delivery.

## THE FUTURE OF GFGP

It is intended that the GFGP Standard becomes an ISO standard. The Kenyan Bureau of Standards (on behalf of the African Organisation for Standardisation) has submitted the GFGP Standard to ISO as a new work item, and it has been accepted. Progressing to ISO is likely to represent a positive step in terms of driving global uptake of GFGP. However, there are a number of factors that need to be considered and challenges to overcome before this can become a reality.

There are currently around 420 to 450 organizations registered on the GGC portal. As this is a new standard, GFGP has yet to reach engagement by a critical mass of organizations or establish a position of long-term sustainability. To reach this point, GFGP requires an increasing number of funders to endorse or advise implementation of the standard and make GFGP-related costs allowable on their grants. This will drive uptake by organizations in the Global North and South and across global health and more widely. The implementation of GFGP by organizations in the Global North is an important development to ensure that GFGP is a truly equitable initiative, regardless of geographical location or organizational type.

## Conclusions

The equitable development of GFGP has created a powerful new standard that addresses challenges within the global health funding model. In our experience of implementing the GFGP Standard, it has proved to be an effective tool for identifying and addressing key organizational risks and for developing institutional financial and grant management capacity across global health partners in Africa, Asia, and Latin America. To fully assess the efficacy of the GFGP Standard, further work is required in implementing the standard more widely and in analyzing the qualitative and quantitative impact of GFGP certification on institutions.

The next steps for GFGP are important ones, but GFGP’s innovative design means that it is well placed to have a long-term future, with the potential to transform the way we give and receive global health funding.

## Notes

***Acknowledgments.*** Members of the NIHR Global Health Research Unit on Genomic Surveillance of Antimicrobial Resistance: Khalil Abudahab, Monica Abrudan, Silvia Argimón, Mihir Kekre, Dawn Muddyman, Ben Taylor, Anthony Underwood, Nicole Wheeler, and Sophia David of the Centre for Genomic Pathogen Surveillance, Big Data Institute, University of Oxford, Old Road Campus, Oxford, United Kingdom and Wellcome Genome Campus, Hinxton, United Kingdom; Pilar Donado-Godoy, Johan Fabian Bernal, Alejandra Arevalo, and Maria Fernanda Valencia of the Colombian Integrated Program for Antimicrobial Resistance Surveillance—Coipars, CI Tibaitatá, Corporación Colombiana de Investigación Agropecuaria (AGROSAVIA), Tibaitatá–Mosquera, Cundinamarca, Colombia; Varun Shamanna, Vandana Govindan, Akshata Prabhu, D. Sravani, M. R. Shincy, Steffimole Rose, and Ravishankar K. N. of the Central Research Laboratory, Kempegowda Institute of Medical Sciences, Bengaluru, India; Anderson O. Oaikhena, Ayorinde O. Afolayan, and Erkison Ewomazino Odih of the Department of Pharmaceutical Microbiology, Faculty of Pharmacy, University of Ibadan, Oyo State, Nigeria; Celia Carlos, Marietta L. Lagrada, Polle Krystle V. Macaranas, Agnettah M. Olorosa, and June M. Gayeta of the Antimicrobial Resistance Surveillance Reference Laboratory, Research Institute for Tropical Medicine, Muntinlupa, the Philippines; Ali Molloy and John Stelling, The Brigham and Women’s Hospital; and Carolin Vegvari, Imperial College London.

The authors thank the following for helpful discussions and contributions: Funmi Oladeji and Babasanmi Osinkolu from the University of Ibadan; Nethravathi Gowda, Rajitha Gowda, and Vandana Govindan from Central Research Laboratory, Kempegowda Institute of Medical Sciences; Marcela Vallejo Beltran, Natalia Andrea Mora Leon, and Edson Manuel Peña Moreno from AGROSAVIA; Sam Musoke, Juan Manuel Palacios, and Rudi Lewin from Humentum; Michael Kilpatrick, Edward Abira, Winfred Muasa, and Vincent Nkundimana from the Global Grant Community; and Gerald Bates from the International Institutions and Donor Assurance team at BDO LLP.

***Disclaimer.*** The views expressed in this publication are those of the authors and not necessarily those of the NHS, the National Institute for Health Research or the Department of Health.

***Financial support.*** This work was supported by Official Development Assistance (ODA) funding from the National Institute for Health Research (grant 16_136_111) and the Wellcome Trust (grant 206194). I. N. O. is supported by an African Research Leader Award from the UK Medical Research Council (MRC) and the UK Department for International Development (DFID) under the MRC/DFID Concordat agreement that is also part of the European and Developing Countries Clinical Trials Partnership 2 program supported by the European Union. This research was commissioned by the National Institute for Health Research using ODA funding. G. K. reports grants from the Wellcome Trust (grant 206194). I.N.O.,H.J.H., and J.J.A. report support from the UK National Institute for Health Research (Global Health Research Unit on Genomic Surveillance of Antimicrobial Resistance Consortium).

***Supplement sponsorship.*** The supplement is sponsored by the UK National Institute for Health Research Global Health Research Unit on Genomic Surveillance of AMR.

***Potential conflicts of interest.*** I.N. O. is a member of the Bill & Melinda Gates Foundation Surveillance Advisors Group (from 2019), has received grants and nonfinancial support from the International Vaccine Institute (Severe Typhoid in Africa Program [SETA] Award to University of Ibadan), and reports grants from UK Medical Research Council and Department for International Development (University of Ibadan—Pathogenic lineages of enteric bacteria in Nigeria MR/L00464X/1). G. K. is a senior advisor to the African Academy of Sciences. G. N. and K. L. R. report travel support grant from UK NIHR. All remaining authors: No reported conflicts of interest. All authors have submitted the ICMJE Form for Disclosure of Potential Conflicts of Interest. Conflicts that the editors consider relevant to the content of the manuscript have been disclosed.
